# Myélolipome de la surrénale: à propos d’un cas

**DOI:** 10.11604/pamj.2017.28.153.13035

**Published:** 2017-10-18

**Authors:** Bouchra Rafiq, Ghizlane El Mghari

**Affiliations:** 1 Service d’Endocrinologie Diabétologie Maladies Métaboliques et Nutrition, Laboratoire PCIM, FMPM, Université Cadi Ayyad, CHU Mohamed VI, Marrakech

**Keywords:** Myélolipome surrénalien, tumeur bénigne, tissu graisseux, Adrenal myelolipoma, benign tumor, fatty tissue

## Abstract

Adrenal myelolipoma is a rare benign non secreting tumor. It is often unexpectedly detected. Pathophysiologically, it is adrenal cortex cell metaplasia into reticuloendothelial cells, resulting from infection, chronic stress or adrenal gland degeneration. The mean age at diagnosis is late forties. Histologically, the tumor is composed of mature fat tissue associated with normal haematopoietic tissue. Tumor echogenicity depends on the predominance of the greasy or myeloid component. Its diagnosis is based on CT scan that identifies the fat percentage. However, these radiological aspects may lead to diagnostic confusion with kidney angiomyolipoma, lipoma and liposarcoma, hence the key role of MRI. Surgical resection of myelolipoma is usually performed and is indicated when tumor is voluminous, compressive or at risk of hemorrhage. We here report the case of AL A, 75 years old, hospitalized for adrenal mass revealed by right back pain radiating to the right hypochondrium, without signs of endocrine hypersecretion. Clinical examination showed sensitivity of the right lumbar fossa. Pheochromocytoma and corticosurrenaloma assessment was without abnormalities, including 24-hours urinary methoxyl derivatives and dexamethasone suppression test. The patient underwent coelioscopy; anatomo-pathological examination showed adrenal myelolipoma.

## Image en médecine

Le myélolipome surrénalien est une tumeur rare, bénigne, non sécrétante, souvent de découverte fortuite. Sa physiopathologie serait une métaplasie des cellules de la corticosurrénale en cellules réticulo-endothéliales en réponse à une infection, à un stress chronique ou à une dégénérescence de la surrénale. La moyenne d’âge de découverte est vers la cinquantaine. Histologiquement, la tumeur est constituée de tissu graisseux mâture, associé à du tissu hématopoïétique normal. Ainsi l'échogénicité de la tumeur est fonction de la prédominance de la composante graisseuse ou myéloïde. Son diagnostique est porté par le scanner qui identifie le contingent graisseux. Toutefois, ces aspects radiologiques peuvent prêter à confusion avec l'angiomyolipome du rein, le lipome et le liposarcome d'où l’intérêt de l'IRM. Habituellement respecté, l'exérèse du myélolipome est indiquée quand il est volumineux, compressif ou présentant un risque hémorragique. Nous rapportons le cas du patient AL A, 75 ans, hospitalisé pour masse surrénalienne, révélée par des lombalgies droites, irradiant vers l'hypocondre droit, sans signes d'hypersécrétion endocrine. L'examen clinique a révélé une sensibilité de la fosse lombaire droite. Le bilan de phéochromocytome et de corticosurrénalome s'est révélé sans anomalies, notamment les dérivés méthoxylés urinaires de 24heures et le freinage minute. Le patient a été opéré par cœlioscopie, avec à l'examen anatomo-pathologique un aspect de myélolipome surrénalien.

**Figure 1:**
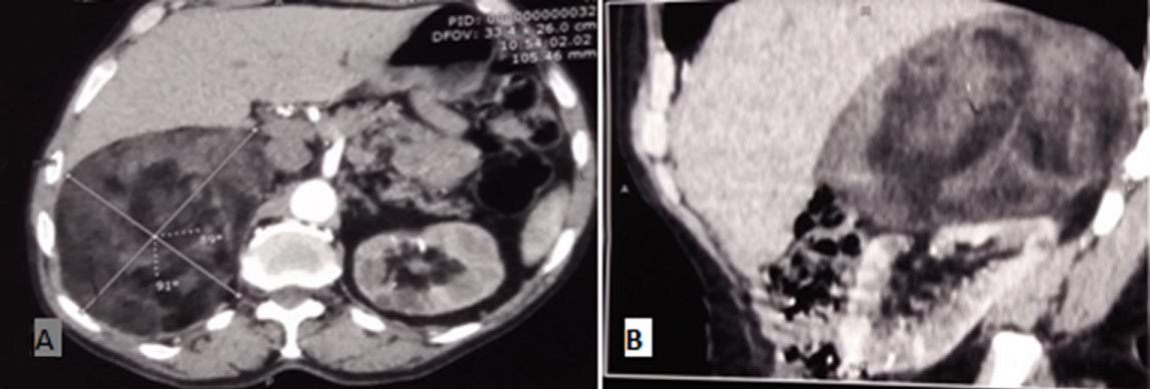
(A, B) scanner abdominal: masse surrénalienne droite mesurant 145*127mm, fibro-adipeuse refoulant le rein et le foie

